# Characteristics and dynamic evolution of inter-industry volatility spillovers in China’s stock market

**DOI:** 10.1371/journal.pone.0330599

**Published:** 2025-09-05

**Authors:** Fusheng Xie, Hongjie Wei

**Affiliations:** 1 Business School, Shanghai Normal University Tianhua College, Shanghai, China; 2 China Aluminum International Trading Group Co., Ltd, Shanghai, China; Northern Border University, SAUDI ARABIA

## Abstract

This study examines the volatility connectedness across 28 sectors in the Chinese stock market, aiming to discern the risk spillovers and their implications for financial security and economic stability. Employing a network connectedness approach, we analyze the volatility connectedness’s characteristics and dynamic evolution among various sectors. The findings indicate that manufacturing industries exhibit a high degree of correlation among themselves and predominantly function as exporters of risk spillovers. Conversely, the financial industry emerges as a primary recipient, characterized by a relatively low correlation to other sectors. During the COVID-19 epidemic, risk correlation within China’s stock market sectors experienced an increase, which, however, did not persist as the epidemic progressed. Furthermore, the conflict between Russia and Ukraine exerted a limited contagion effect on China’s stock market risks. These insights offer valuable guidance for China in managing economic and financial risks more effectively.

## 1. Introduction

Accurately monitoring the interconnectedness of risks across diverse sectors is imperative for safeguarding financial stability, fostering sustainable economic growth, and mitigating the spillover of sector-specific risks during periods of significant disruption. This study investigates the industry-level risk spillovers within China’s stock market by examining fluctuations in sectoral indices. By doing so, it aims to elucidate the distinct roles that various industries play in the transmission of systemic risk, thereby providing valuable insights for policymakers to craft effective industry-specific risk management strategies and preventive measures.

In recent years, a series of major emergencies have occurred with increasing frequency, exerting a profound impact on the global economic environment and financial markets. From the global financial crisis of 2008 (Giglio et al., 2016) [1], the Sino-U.S. trade friction in 2018 (Kim M., 2019) [2], to the Russia-Ukraine conflict in 2022 (Orhan E., 2022) [3], these events have not only reshaped the international political landscape but also severely impacted the Chinese economy. Amidst these developments, a critical question arises: does heterogeneity in risk spillover exist among various economic sectors? Furthermore, which sectors act as exporters of risk, and which become recipients? Moreover, do these roles shift dynamically over time? Accurately monitoring the correlation structure of risks across sectors is crucial for maintaining financial security, achieving economic stability and sustainable development, and preventing sectoral risk spillovers during significant events (Wu et al., 2019; Lou et al., 2024) [4,5].

The current macroeconomic landscape stands at a critical juncture of economic transition and industrial restructuring. Analyzing cross-industry risk spillover effects facilitates objective risk assessment in strategic sectors. This paper aims to explore the characteristics of industry risk spillover in China’s stock market by examining the fluctuations of industry indices. This study employs a multidimensional analytical framework encompassing both static and dynamic perspectives, focusing on three core dimensions: total risk effect, directional spillover effect, and net spillover effect. Specifically, this study focuses on the high-dimensional risk contagion network spanning 28 industrial sectors, aiming to reveal the roles of each industry in the chain of systemic risk transmission and provide theoretical support for the formulation of industry policies and risk prevention measures.

In October 2020, the Central Committee of the Communist Party of China proposed in its recommendations for the formulation of the 14th Five-Year Plan for National Economic and Social Development and the Vision for 2035 to strengthen economic security risk early warning, prevention, and control mechanisms to ensure security and controllability in key areas such as important industries, infrastructure, strategic resources, and major science and technology projects. With the transformation and upgrading of the economic structure and the adjustment and optimization of the financial structure, systemic risk has gradually exhibited new characteristics and trends of cross-industry contagion (Chang et al., 2024; Li et al., 2024) [6,7]. Against this backdrop, what roles do China’s industries play in systemic risk transmission? What are the specific characteristics of industry risk spillover? Is the financial industry a source of risk spillover? Answering these questions is crucial for enhancing risk early warning capabilities and maintaining sustained and stable economic development (Bisias et al., 2012; Benoit et al., 2017) [8,9].

The concept of systemic risk has spurred a substantial body of research (Haldane & May, 2011; Raddant & Kenett, 2021) [10,11]. This study centers on the examination of systemic risk within the financial domain. As economic and financial integration deepens, systemic risk has commenced propagating through new pathways among diverse sectors and industries (Baruník & Křehlík, 2018) [12]. To elucidate these characteristics of risk correlation, this paper employs the DY model (Diebold & Yilmaz, 2012) [13] and its variants, including the LASSO-based DY model (Jia & Du, 2024) [14] and the elastic network shrinkage technology-based DY model (Gross & Siklos, 2022) [15], to explore the high-dimensional network of industry risk spillover in China’s stock market.

The innovation of this paper lies in its systematic literature review and comparative analysis of the risk spillover effects between international and domestic financial markets, as well as an in-depth exploration of the structural characteristics and dynamic changes of risk spillovers across 28 industries in China’s stock market. By incorporating LASSO and elastic network shrinkage technologies, this paper effectively addresses the “ curse of dimensionality “of the VAR model, thereby enhancing the accuracy and reliability of the research findings. This provides a clearer revelation of the characteristics and dynamic evolution of industry risk spillover effects. Furthermore, the paper explores the risk contagion mechanism from an event-driven risk perspective.

The structure of the remainder of this paper is organized as follows: the subsequent section presents a comprehensive literature review, the third section delineates the data employed in this study and offers preliminary analyses, and the fourth section reports empirical findings and discusses these results in the context of the aforementioned research questions. Finally, the concluding section summarizes the study and provides closing remarks.

## 2. Literature review

For a prolonged period, the cross-market contagion of financial risks has been a central concern in systemic risk research. Shen et al. (2020) [16] focus on the sectoral connectedness within the Chinese stock market, revealing volatility spillover effects and risk contagion patterns. Qiao et al. (2020) [17] delve into the industry-specific volatility spillover networks in China’s stock market, particularly highlighting the role of certain service sectors during the COVID-19 outbreak. Özdurak et al. (2020) [18] investigate the impact of China’s economic slowdown on financial and energy markets, emphasizing the cross-asset contagion mechanisms between the US and Chinese stock markets. In addition, Vlasova et al. (2022) [19] explore the volatility spillovers between the Russia–India–China triad and the United States, shedding light on the economic linkages and mutual influence of financial markets. Jufri et al. (2022) [20] studied the spillover effects of the Islamic stock indexes of the US and China on the ASEAN Islamic stock index during the COVID-19 pandemic, identifying asymmetric spillover effects. Chen et al. (2024) [21] analyze the dynamic interdependence structure and risk spillover effects between the US and Chinese stock markets, emphasizing the role of different sectors in the interdependent structure. Mohanty et al. (2023) [22] examine the relationship between foreign exchange rate fluctuations and stock market indices in India, while Kakran et al. (2023) [23] use the Diebold–Yilmaz index model to measure volatility spillovers among APEC stock markets. Although some studies have focused on the role of specific sectors in risk contagion, the research on the heterogeneity of risk transmission between different industries is not comprehensive enough. For instance, different industries may exhibit varying responses and contagion capacities when faced with the same external shocks.

The existing literature on financial risk contagion primarily utilizes low-dimensional frameworks, which may fail to capture the complex dynamics between multiple institutions and markets, potentially leading to biased research findings. A substantial body of literature has delved into the return linkage of financial assets (Brunetti et al., 2019) [24], volatility spillover (Shahzad et al.,2021) [25], and tail risk resonance (He et al.,2021; Zhang & Chen, 2022) [26,27] from various perspectives. Existing empirical research primarily employs four methodological approaches: the GARCH family model analysis (Rejeb & Arfaoui, 2016) [28], the Copula function analysis (Aloui et al., 2011) [29], the generalized Granger causality test (Hong et al., 2009) [30], and the spillover index method (Antonakakis & Badinger, 2016; Yao et al.,2022) [31,32]. However, the majority of these studies are constrained within the traditional low-dimensional framework. The research scope is narrow, making it challenging to capture the intricate risk contagion dynamics between multiple institutions and diverse markets, which may introduce significant bias into the research findings (Barigozzi & Hallin, 2017) [33]. This limitation arises from the potential issue of missing variables in the selection of samples in low-dimensional networks, leading to biased model specifications.

With the continuous evolution of modern econometric techniques, addressing the “curse of dimensionality” in high-dimensional models has become a critical focus in financial research. Traditional approaches, such as Vector Autoregressive (VAR) and Generalized Autoregressive Conditional Heteroskedasticity (GARCH) models, have long been employed to analyze risk contagion and volatility spillovers. However, these methods often struggle with high-dimensional data due to their inherent limitations in handling large numbers of variables, leading to overfitting and reduced predictive accuracy. To overcome these challenges, scholars have increasingly turned to advanced techniques such as LASSO (Least Absolute Shrinkage and Selection Operator) (Demirer et al., 2018) [34] and elastic net regularization (Gross & Siklos, 2020) [15], which have demonstrated remarkable success in analyzing complex, high-dimensional datasets.

The LASSO method effectively reduces model complexity and enhances interpretability by introducing a penalty term that shrinks less important coefficients to zero. This approach has significant advantages in identifying key drivers of risk contagion within high-dimensional networks. Building on LASSO, the Elastic Net technique combines the strengths of LASSO and Ridge Regression, offering a more robust solution for multicollinearity and variable selection. This method is particularly suitable for high-dimensional model estimation based on rolling windows, enabling dynamic analysis of risk spillovers over time.

By accurately identifying key risk transmitters and receivers within financial systems, LASSO and Elastic Net techniques empower practitioners to design targeted risk management strategies. For example, financial institutions can prioritize monitoring and mitigating risks in sectors identified as major sources of volatility spillovers. Regulators can also leverage these insights to implement sector-specific risk controls, such as capital buffers or stress testing requirements, thereby enhancing the resilience of the financial system.

From the standpoint of research objects, the existing literature predominantly focuses on the cross-market contagion of international financial risks and the risk contagion within financial markets. However, there is a pressing need to strengthen research on the interactions between financial markets and real industries, particularly the risk spillover characteristics and dynamic changes between subsectors. In terms of research methods, the traditional low-dimensional framework may introduce significant errors in the research conclusions due to the potential presence of missing variables. This paper leverages the elastic net regularization technique and the LASSO method to analyze the risk spillover within China’s stock market between industries, thereby enhancing the accuracy and efficacy of the analysis.

Some literature has examined the impact of major emergencies on the economic or financial system. Alfaro et al. (2020) [35] and Han (2022) [36] have investigated the impact of the novel coronavirus pandemic on U.S. financial markets and China’s economy. Jiang et al. (2021) [37] and Ye et al. (2021) [38] have analyzed the risk spillover effects of the new coronavirus on global financial markets and between European, American, and Chinese stock markets. Li et al. (2020) [39] have studied the spillover effect of the Sino-U.S. trade friction on China’s financial market. Moreover, some scholars have comprehensively analyzed the impact of various major events such as financial crises, the novel coronavirus pandemic, stock market crashes, and other significant events on the inter-industry and inter-country financial markets (Yang et al.,2020; Li et al., 2023; Choi and Yoon, 2023) [40–42]. These studies have found that major emergencies will bring significant impact and structural changes to the economic and financial system. However, there is still a paucity of literature that explores the risk spillover effect of the new coronavirus pandemic on Chinese industries. Additionally, there is a scarcity of literature that investigates the risk spillover effect of the Russia-Ukraine international conflict on Chinese industries.

In light of these gaps, this paper aims to bridge the divide by utilizing advanced econometric methods to analyze the risk spillover dynamics within the Chinese stock market across various industries. By employing the elastic net technology, and the LASSO method, this study seeks to provide a more accurate and comprehensive understanding of the complex risk contagion mechanisms that operate within and between these sectors. The findings of this research are expected to contribute to the development of effective risk management strategies and policy recommendations that can mitigate the adverse effects of such events on the Chinese economy and financial markets.

## 3. Data overview and methods

### 3.1. Data

This study adopts the CITIC Securities industry classification system, utilizing first-tier sector indices obtained from the Wind Financial Database. Based on the 2020 classification framework encompassing 30 primary sectors, we exclude two industry indices: the composite index (as it cannot clearly identify specific industries) and the newly added composite financial industry index (due to insufficient historical data). Our sample selection criteria yielded 28 representative industrial sectors, including Petroleum and petrochemicals, Coal Mining, Non-ferrous Metals, Power and utilities, Steel, Base chemical, Construction, Building materials, Light manufacturing, Mechanical industry, Power Equipment and New Energy, National defense, Automobile, Commercial retail, Customer service, Household appliances, Textile and apparel, pharmaceutical, Food and drink, Agriculture and forestry, Bank, Non-bank financial, Real estate, Transportation, Electronic, Communications, Computer and Media. The dataset spans January 1, 2005 to May 31, 2023 (n = 4,472 daily observations), containing comprehensive market metrics including open, high, low, and closing prices. For ease of reference, the sector name and abbreviation are shown in Table 1.

**Table 1:** Sector indexes.

Serial number	sector name	abbreviation
1	Petroleum and petrochemicals	PP
2	Coal Mining	Coal
3	Non-ferrous Metals	NFM
4	Power and utilities	PU
5	Steel	Steel
6	Base chemical	Bchem
7	Construction	Const
8	Building materials	BM
9	Light manufacturing	LMF
10	Mechanical industry	Mech
11	Power Equipment and New Energy	PENE
12	National defense	ND
13	Automobile	Auto
14	Commercial retai	CommR
15	Customer service	CS
16	Household appliances	Happ
17	Textile and apparel	TA
18	pharmaceutical	Pharm
19	Food and drink	FD
20	Agriculture and forestry	AF
21	Bank	Bank
22	Non-bank fiancial	NBF
23	Real estate	Rest
24	Transportation	Trans
25	Electronic	Elec
26	Communications	Comm
27	Computer	Comp
28	Media	Media

**Note:** CITIC Securities Industry Classification

To investigate the volatility connectedness, we first define the daily volatility for each index following Garman and Klass (1980) [43], which is daily range-based realized volatility.


$$\sigma_{it}^{2}=0.511{\left(H_{it}-L_{it}\right)}^{2}-0.019\left[\left(C_{it}-O_{it}\right)\left(H_{it}+L_{it}-2O_{it}\right)-2\left(H_{it}-O_{it}\right)\left(L_{it}-O_{it}\right)\right]-0.383{\left(C_{it}-O_{it}\right)}^{2}$$ (1)

where $H_{it},L_{it},O_{it}$ and $C_{it}$ respectively the logs of daily high, low, opening, and closing values for sector *i* on day *t*. Usually, the realized volatility is the annualized standard deviation. Hence, the annualized volatility is used in the following.

Table 2 shows the summary statistics of sector volatilities during the whole period. As Shen et al. (2022) [16] have been acquired from sector classification by Shenyin & Wanguo securities, we also find that for all volatility series, the mean value is larger than its median value, suggesting that the tail is on the right, although the data differ. The kurtosis values are much greater than 3, and the skewness is positive, also implying that all series are significantly leptokurtic, which also supports the findings of Shen et al. (2022) [16].

**Table 2:** Descriptive statistics of sector volatilities.

	Mean	Median	Max	Min	Std	Skew.	Kurt.
**PP**	24.25	18.72	135.08	0.04	16.58	1.49	5.90
**Coal**	29.81	24.93	130.55	0.05	18.45	1.34	5.25
**NFM**	30.53	25.38	143.77	0.20	19.33	1.21	4.74
**PU**	22.50	16.66	157.69	0.22	17.11	1.78	7.75
**Steel**	26.20	20.96	149.17	0.24	17.61	1.63	6.52
**Bchem**	26.38	19.76	133.33	0.05	18.55	1.29	4.86
**Const**	25.58	19.74	152.13	0.66	18.33	1.59	6.28
**BM**	28.45	22.13	139.88	0.26	19.09	1.32	4.96
**LMF**	26.75	19.84	137.53	0.09	18.90	1.28	4.91
**Mech**	22.50	16.66	157.69	0.22	17.11	1.78	7.75
**PENE**	28.64	23.26	141.21	0.81	18.94	1.19	4.79
**ND**	31.57	26.78	151.66	0.23	19.70	1.39	6.01
**Auto**	27.57	21.42	135.55	0.07	18.94	1.24	4.70
**CommR**	26.19	18.40	152.31	0.45	19.63	1.33	4.91
**CS**	29.45	23.91	139.80	0.41	19.34	1.29	4.99
**Happ**	27.00	21.95	138.94	0.13	16.95	1.41	5.54
**TA**	26.15	18.01	132.90	0.17	20.26	1.33	4.64
**Pharm**	26.59	20.90	145.64	0.37	18.32	1.25	4.90
**FD**	26.20	21.92	121.73	0.77	15.85	1.32	5.36
**AF**	28.98	22.84	158.37	0.23	20.08	1.30	4.98
**Bank**	22.13	17.20	135.16	0.00	16.06	1.79	7.69
**NBF**	28.68	23.05	181.21	0.00	19.06	1.61	7.05
**Rest**	28.85	22.66	140.51	0.45	19.79	1.24	4.64
**Trans**	24.26	17.56	146.42	0.06	17.94	1.59	6.26
**Elec**	29.41	23.44	145.06	0.38	19.04	1.36	5.38

### 3.2. Methodology

This section introduces the connectedness approach used in this paper. Consider the vector autoregressive model, which follows directly from the familiar notion of a variance decomposition associated with an m-variable vector autoregression.


$$X_{t}=\sum_{i=1}^{p}\Phi_{i}X_{t-i}+\varepsilon_{t}$$ (2)

where $X_{t}={\left(x_{1t},x_{2t},\ldots ,x_{mt}\right)}^{\prime }$ is a m×1 vector of jointly determined dependent variables, and $\left\{\Phi_{i}:i=1,2,...,p\right\}$ are m×m coefficient matrices.

Concerning Pesaran and Shin (1998) [44] and Lütkepohl (2005) [45], the following standard assumptions are given:

**Assumption 3.1** $E(\varepsilon_{t})=0$, $E(\varepsilon_{t}{\varepsilon_{t}}^{\prime })=\Sigma$ for all *t*, where $\sum =\left\{\sigma_{ij},i,j=1,2,...,m\right\}$ is a m×m positive definite matrix, $E(\varepsilon_{t}{\varepsilon_{t\prime }}^{\prime })=0$ for all t≠t′.

**Assumption 3.2** All the roots of $\left|I_{m}-\sum {\mathrm{\backslash nolimits}}_{i=1}^{p}\Phi_{i}Z^{i}\right|=0$ fall outside the unit circle.

**Assumption 3.3** $\;X_{t-1},X_{t-2},...,X_{t-p},t=1,2,....T$, are not perfectly collinear.

Under Assumption 3.2, $X_{t}$ would be covariance-stationary, and (2) can be rewritten as the infinite moving average representation,


$$X_{t}=\sum_{i=1}^{\infty }A_{i}\varepsilon_{t-i},t=1,2,...,T$$ (3)

$A_{i}$ are m×m coefficient matrices, and $A_{i}$ can be obtained using the following recursive relations:


$$A_{i}=\sum_{k=1}^{p}\Phi_{k}A_{i-k},\;i=1,2,...$$ (4)

with $A_{0}=I_{m}$ and $A_{i}=0$ for i<0.

When $\varepsilon_{t}$ has a multivariate normal distribution, by Koop et al. (1996) [46], we have


$$GI_{x}(h,\delta_{j},\Omega_{t-1})=E(X_{t+h},\varepsilon_{t}=\delta_{j},\Omega_{t-1})-E(X_{t+h},\Omega_{t-1})=A_{h}\Sigma e_{j}\sigma_{jj}^{-1}\delta_{j}$$

By setting $\delta_{j}=\sqrt{\sigma_{jj}}$, we obtain the scaled generalized impulse response function by the error of the optimal *h*-step forecast:


$$\Psi_{j}^{g}\left(h\right)=\sigma_{jj}^{-1/2}A_{h}\Sigma e_{j}\;h=0,1,2,...$$ (5)

which measures the effect of a standard shock to the *j*th equation at time *t* on expected values of *X*_*t+h*_.

The above generalized impulses can also be used in the derivation of the forecast error variance decompositions, defined as the proportion of the h-step ahead forecast error variance of variable *i* which is accounted for by the innovations in variable *j* in the VAR. The KPPS (Koop, Pesaran, and Potter,1996) [46] *h*-step-ahead forecast error variance decompositions:


$$\theta_{ij}^{g}(h)=\frac{\sigma_{jj}^{-1}\sum {\mathrm{\backslash nolimits}}_{k=0}^{h-1}{\left({e_{i}}^{\prime }A_{k}\Sigma e_{j}\right)}^{2}}{\sum {\mathrm{\backslash nolimits}}_{k=0}^{h-1}{e_{i}}^{\prime }A_{k}\Sigma {A\prime }_{k}e_{i}}$$ (6)

In order to utilize the information available in the variance decomposition matrix for the calculation of the spillover index, we normalize each row of the variance decomposition matrix, thus, we obtain:


$$d_{ij}^{g}(h)=\frac{\theta_{ij}^{g}(h)}{\sum {\mathrm{\backslash nolimits}}_{j=1}^{m}\theta_{ij}^{g}(h)}$$ (7)

So, $\sum {\mathrm{\backslash nolimits}}_{j=1}^{m}d_{ij}^{g}(h)=1,\sum {\mathrm{\backslash nolimits}}_{i,j=1}^{m}d_{ij}^{g}(h)=m.$

Using the Generalized Forecast Error Variance Decomposition (GFEVD), we construct the total connectedness index as follows:


Tg(h)=∑\nolimitsi,j=1\hfilli≠j\hfillmdijg(h)∑\nolimitsi,j=1mdijg(h)=∑\nolimitsi,j=1\hfilli≠j\hfillmdijg(h)m (8)

The Generalized Forecast Error Variance Decomposition (GFEVD) is a tool used in time series analysis to understand how different variables contribute to the uncertainty in our forecasts. Imagine you are trying to predict the weather, and you want to know how much each factor—like temperature, humidity, and wind speed—contributes to the overall uncertainty in your forecast. GFEVD helps you break down these contributions and see which factors are the most important. The aggregate spillover index measures the contribution of spillovers from cross-sectoral volatility shocks to the total forecast error variance.

All connectedness measures developed by Diebold and Yilmaz(2014) [47] can be derived from GFEVD as follows:


Fi←.g(h)=∑\nolimitsj=1\hfilli≠j\hfillmdijg(h)×100 (9)

T.←ig(h)=∑\nolimitsj=1\hfilli≠j\hfillmdjig(h)×100 (10)

$${N_{i}}^{g}(h)={T_{.\leftarrow i}}^{g}(h)-{F_{i\leftarrow .}}^{g}(h)$$ (11)

$${P_{ij}}^{g}(h)=\left(\frac{d_{ij}^{g}(h)}{\sum {\mathrm{\backslash nolimits}}_{k=1}^{m}d_{ik}^{g}(h)}-\frac{d_{ji}^{g}(h)}{\sum {\mathrm{\backslash nolimits}}_{k=1}^{m}d_{jk}^{g}(h)}\right)\times 100=d_{ij}^{g}(h)-d_{ji}^{g}(h)$$ (12)

${F_{i\leftarrow .}}^{g}(h)$ ( ${T_{.\leftarrow i}}^{g}(h)$) is the volatility spillovers received by sector *i* from (to) all other sector *j*, representing the total directional connectedness from (to) others. Is the difference between ${T_{.\leftarrow i}}^{g}(h)$ and ${F_{i\leftarrow .}}^{g}(h)$, representing the net total directional connectedness, which identifies whether sector *i* is a net transmitter or a net receiver of shocks. Sector *i* is a net transmitter of shocks when the impact sector *i* has on all others is larger than the influence all others have on sector *i*; otherwise, sector *i* is a net receiver of shocks. Finally, The net two-by-two volatility spillover (${P_{ij}}^{g}(h)$) which represents the net pairwise directional connectedness and measures if sector *i* is influencing sector *j* more and vice versa.

In estimating the high-dimensional VAR model, this paper addresses the ‘curse of dimensionality’ problem, this paper draws on the research methods of Gross and Siklos (2020) [15] and Gabauer et al. (2020) [48] to solve the following optimization problem:


$$\hat{\beta }=\underset{\beta }{\mathrm{\backslash arg}\min}\left\{\sum {\mathrm{\backslash nolimits}}_{t=1}^{T}{\left[X_{it}-\sum {\mathrm{\backslash nolimits}}_{k=1}^{m}\left(\beta_{ki}^{\prime }X_{it-k}\right)\right]}^{2}+\gamma \sum {\mathrm{\backslash nolimits}}_{k=1}^{m}\left[\left(1-\delta \right)\left|\beta_{ki}\right|+\delta {\left|\beta_{ki}\right|}^{2}\right]\right\},\;0\leq \delta \leq 1$$

In this paper, let δ=0, that is, the LASSO shrinkage technique is used to estimate the coefficients, and then the elastic network method is used to test the robustness of the full sample.

**Fig 1:**
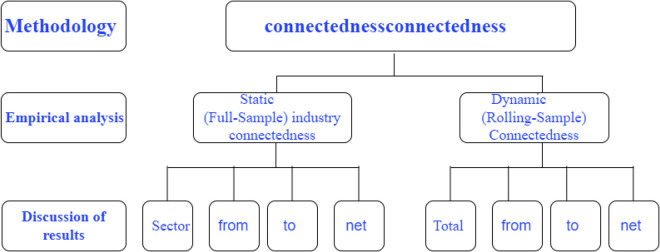
Technical framework diagram. **Note:** sectors(sectors connectedness),from(from- connectedness), to(to- connectedness), net(net- connectedness), Total(Total connectedness).

Fig 1 is the technical framework diagram of this study.

## 4. Benchmark results I: Static (Full-Sample) industry connectedness

### 4.1. Measuring connectedness

We examine sector index return volatility connectedness using the framework of Diebold and Yilmaz (2014) [47]. In particular, for the benchmark results reported in Sections 4 and 5, we proceed as follows:

(1) We use a VAR(4) approximating model, estimating VAR models using the LASSO method.
(2) We identify the estimated VAR using the generalized approach of Pesaran and Shin (1998) [44], and then examine variance decompositions at horizon H = 10 days.
(3) We summarize the variance decomposition matrix using connectedness statistics (Total Spillovers, Directional Spillovers, Net Spillovers).

In Table 3, V*i* (*i = *1,2,…*,m*) represents the volatility of the *i*th sector. $d_{ij}^{g}(h)$ is the sector *i*’s *h*-step-ahead generalized forecast error variance due to the shocks from sector *j*. ${F_{i\leftarrow .}}^{g}(h)$ and ${T_{.\leftarrow i}}^{g}(h)$ stand for from-connectedness and to-connectedness, respectively. ${N_{i}}^{g}(h)$ stands for net-connectedness. The significance of Table 3 lies in its ability to provide a comprehensive overview of the interconnectedness and risk transmission dynamics among sectors. By quantifying the directional and net spillover effects, the table helps identify key sectors that act as major sources or recipients of volatility shocks.

**Table 3:** Connectedness Table.

	V1	V2	⋯	Vm	${F_{i\leftarrow .}}^{g}(h)$
V1	$d_{11}^{g}(h)$	$d_{12}^{g}(h)$	⋯	$d_{1m}^{g}(h)$	∑\nolimitsj=1\hfill1≠j\hfillmd1jg(h)
V2	$d_{21}^{g}(h)$	$d_{22}^{g}(h)$	⋯	$d_{2m}^{g}(h)$	∑\nolimitsj=1\hfill2≠j\hfillmd2jg(h)
⋮	⋮	⋮	⋱	⋮	⋮
Vm	$d_{m1}^{g}(h)$	$d_{m2}^{g}(h)$	⋯	$d_{mm}^{g}(h)$	∑\nolimitsj=1\hfillm≠j\hfillmdmjg(h)
${T_{.\leftarrow i}}^{g}(h)$	∑\nolimitsj=1\hfill1≠j\hfillmdj1g(h)	∑\nolimitsj=1\hfill2≠j\hfillmdj2g(h)	⋯	∑\nolimitsj=1\hfillm≠j\hfillmdjmg(h)	
${N_{i}}^{g}(h)$	${T_{.\leftarrow 1}}^{g}(h)$–${F_{1\leftarrow .}}^{g}(h)$	${T_{.\leftarrow 2}}^{g}(h)$–${F_{2\leftarrow .}}^{g}(h)$	⋯	${T_{.\leftarrow m}}^{g}(h)$–${F_{m\leftarrow .}}^{g}(h)$	

**Note:** Definition of connectedness measures. Vi represents the volatility of the *i*-th sector. The KPPS (Koop, Pesaran, and Potter,1996) [46] h-step-ahead forecast error variance decompositions: $\theta_{ij}^{g}(h)=\frac{\sigma_{jj}^{-1}\sum {\mathrm{\backslash nolimits}}_{k=0}^{h-1}{\left({e_{i}}^{\prime }A_{k}\Sigma e_{j}\right)}^{2}}{\sum {\mathrm{\backslash nolimits}}_{k=0}^{h-1}{e_{i}}^{\prime }A_{k}\Sigma {A\prime }_{k}e_{i}}$, $d_{ij}^{g}(h)=\frac{\theta_{ij}^{g}(h)}{\sum {\mathrm{\backslash nolimits}}_{j=1}^{m}\theta_{ij}^{g}(h)}$ is the fraction of industry *i*’s *H*-step-ahead generalized forecast error variance due to the shocks from sector *j*. ${F_{i\leftarrow .}}^{g}(h)$, ${T_{.\leftarrow i}}^{g}(h)$ and ${N_{i}}^{g}(h)$ stand for from-connectedness, to-connectedness and net-connectedness, respectively. ${F_{i\leftarrow .}}^{g}(h)$ (${T_{.\leftarrow i}}^{g}(h)$) is the volatility spillovers received by sector *i* from (to) all other sector *j*, representing the total directional connectedness from (to) others. ${N_{i}}^{g}(h)$ is the difference between ${T_{.\leftarrow i}}^{g}(h)$ and ${F_{i\leftarrow .}}^{g}(h)$, representing the net total directional connectedness, which identifies whether sector *i* is a net transmitter or a net receiver of shocks.

The volatility connectedness matrix is constructed using pairwise directional connectedness measures across sectors. The matrix elements fall within a range of 1.28% to 17.14%. Diagonal elements of the matrix denote each sector’s self-connectedness. As indicated in Table 4, the Light Manufacturing sector (LMF) exhibits the lowest self-volatility connectedness at 6.8%, suggesting a relatively minor internal impact. This sector is subject to influences from multiple departments and is particularly sensitive to external shocks.

**Table 4:** Sector Connectedness Table.

	PP	Coal		NFM	PU	Steel	Bchem			Const	BM		LMF		Mech			PENE		ND		Auto	CommR		CS
PP	9.58	3.3		3.3	4.12	3.24	4.37			3.84	3.92		4.29		4.12			3.81		2.51		3.79	3.82		2.79
Coal	4.29	13.12		4.66	3.67	4.86	3.85			3.77	3.72		3.69		3.67			3.17		2.34		3.53	3.34		2.36
NFM	3.51	3.96		10.23	3.49	4.02	4.75			3.47	3.93		4.43		3.49			4.11		2.93		3.95	3.67		2.72
PU	3.44	2.46		2.68	8.27	3.09	4.45			3.98	3.74		4.38		8.24			3.86		2.62		3.79	3.88		2.79
Steel	3.55	4.23		4.16	4.01	10.76	4.26			3.91	4.04		4.23		4.01			3.42		2.59		3.65	3.69		2.5
Bchem	3.26	2.27		3.33	4.03	2.93	6.93			3.47	4.09		5		4.02			4.75		2.93		4.33	4.25		3.16
Const	3.52	2.68		2.89	4.26	3.25	4.15			8.62	4.43		4.66		4.26			3.68		2.51		3.7	4.09		2.92
BM	3.28	2.49		3.12	3.83	3.12	4.63			4.17	7.78		4.8		3.83			4.13		2.73		4.1	4.11		3.05
LMF	3.17	2.22		3.09	3.91	2.91	4.95			3.83	4.19		6.8		3.91			4.6		3.12		4.46	4.24		3.04
Mech	3.43	2.46		2.68	8.24	3.09	4.45			3.98	3.74		4.38		8.27			3.86		2.62		3.79	3.88		2.79
PENE	3.07	2.07		3.23	3.81	2.59	5.24			3.32	3.95		5.09		3.81			7.71		3.01		4.59	4.16		3.16
ND	2.88	2.12		3.17	3.67	2.76	4.48			3.22	3.66		4.85		3.67			4.26		10.88		4.03	3.85		3.01
Auto	3.12	2.34		3.18	3.79	2.81	4.84			3.38	4.01		5.07		3.79			4.72		2.93		8.02	4.04		3.06
CommR	3.08	2.17		2.78	3.77	2.75	4.57			3.7	3.89		4.62		3.77			4.13		2.67		3.94	7.74		3.35
CS	2.92	1.95		2.68	3.4	2.31	4.27			3.33	3.72		4.17		3.4			4.02		2.62		3.81	4.28		10.63
Happ	2.99	2.27		2.71	3.51	2.54	4.01			3.32	3.97		4.11		3.51			3.88		2.28		4.38	3.81		3.22
TA	3.12	2.06		2.7	3.97	2.58	4.8			3.78	3.99		4.73		3.97			4.24		2.8		4.02	4.95		3.34
Pharm	2.92	1.97		2.62	3.71	2.56	4.8			3.27	3.85		4.56		3.71			4.31		2.66		4.02	4.32		3.63
FD	2.98	2.47		2.78	3.44	2.64	4.11			3.12	3.66		3.93		3.44			3.59		2.31		3.72	3.87		3.59
AF	3	2.11		2.87	3.77	2.47	4.61			3.28	3.71		4.32		3.77			4.25		2.88		3.67	4.22		3.34
Bank	4.01	3.09		2.44	3.16	3.06	2.85			3.53	3.26		3.3		3.15			2.58		1.96		3.21	2.99		2.47
NBF	3.45	3.33		3.11	3.22	3.06	3.09			3.74	3.56		3.57		3.22			2.97		2.37		3.4	3.46		2.21
Rest	3.2	2.82		2.75	3.57	2.85	3.68			4.48	4.32		4		3.57			3.44		2.38		3.58	3.98		3.24
Trans	3.47	2.52		2.79	4.04	3.12	4.21			4.08	4.02		4.52		4.04			3.66		2.57		4.04	4.55		3.26
Elec	2.73	1.99		2.88	3.41	2.4	4.85			3.18	3.66		4.83		3.41			4.71		3.09		4.13	4.01		3.14
Comm	2.99	2.12		2.76	3.46	2.62	4.35			3.27	3.57		4.68		3.46			4.25		3		3.88	4.03		3
Comp	2.65	1.83		2.59	3.49	2.35	4.55			3.29	3.57		4.65		3.49			4.28		3.1		3.84	4.2		3.15
Media	2.79	1.75		2.57	3.57	2.36	4.26			3.33	3.52		4.39		3.57			4.19		2.69		3.65	4.52		3.43
TO	86.83	67.04	80.53		104.33	78.34	117.42			97.04	103.69		119.23		104.29			106.85		72.22		104.99	108.21		81.75
NET	−3.59	−19.84	−9.24		12.61	−10.91	24.36			5.65	11.47		26.03		12.57			14.56		−16.9		13.02	15.96		−7.62
	Happ	TA	Pharm		FD	AF		Bank	NBF			Rest		Trans		Elec	Comm		Comp		Media			FROM	
PP	2.84	3.89	3.34		2.6	3.08		2.29	2.39			2.87		4.02		3.09	3.16		2.83		2.79			90.42	
Coal	2.61	3.17	2.76		2.58	2.7		2.34	2.92			3.2		3.64		2.73	2.78		2.38		2.16			86.88	
NFM	2.69	3.6	3.11		2.53	3.1		1.59	2.31			2.71		3.46		3.39	3.14		2.96		2.75			89.77	
PU	2.76	4.16	3.49		2.45	3.2		1.57	1.86			2.66		3.9		3.18	3.06		3.07		2.97			91.73	
Steel	2.63	3.48	3.16		2.51	2.8		2.05	2.34			2.79		3.96		2.94	3.04		2.74		2.56			89.24	
Bchem	2.86	4.51	4.03		2.63	3.59		1.32	1.65			2.48		3.67		4.07	3.53		3.65		3.25			93.07	
Const	2.86	4.23	3.36		2.43	3.06		1.91	2.37			3.45		4.28		3.21	3.14		3.13		2.97			91.38	
BM	3.12	4.24	3.67		2.59	3.28		1.69	2.16			3.17		3.94		3.49	3.24		3.24		2.98			92.22	
LMF	2.89	4.38	3.81		2.52	3.32		1.51	1.88			2.67		3.89		4	3.72		3.69		3.28			93.2	
Mech	2.76	4.16	3.49		2.45	3.2		1.57	1.86			2.66		3.9		3.18	3.06		3.07		2.97			91.73	
PENE	2.96	4.31	3.95		2.48	3.54		1.28	1.69			2.48		3.43		4.3	3.7		3.71		3.4			92.29	
ND	2.51	4.05	3.46		2.3	3.45		1.4	1.91			2.43		3.42		3.93	3.67		3.8		3.15			89.12	
Auto	3.38	4.15	3.78		2.58	3.14		1.58	1.98			2.65		3.86		3.88	3.49		3.4		3.03			91.98	
CommR	2.9	5	3.93		2.61	3.52		1.46	1.96			2.88		4.28		3.64	3.52		3.67		3.68			92.26	
CS	3.2	4.29	4.28		3.22	3.53		1.64	1.68			2.96		3.99		3.6	3.25		3.39		3.46			89.37	
	Happ	TA	Pharm		FD	AF		Bank	NBF			Rest		Trans		Elec	Comm		Comp		Media			FROM	
Happ	10.75	3.79	3.81		3.68	3.23		2.22	2.47			2.92		3.75		3.53	3.22		3.19		2.96			89.25	
TA	2.89	7.58	3.96		2.41	3.69		1.32	1.7			2.74		3.99		3.68	3.52		3.85		3.63			92.42	
Pharm	3.11	4.39	8.62		3.38	3.64		1.49	1.8			2.49		3.57		4.08	3.46		3.72		3.32			91.38	
FD	4.02	3.54	4.59		12.15	3.39		2.03	2.41			2.74		3.66		3.34	3.08		2.83		2.56			87.85	
AF	2.96	4.52	3.97		2.8	10.71		1.35	1.61			2.54		3.66		3.58	3.32		3.37		3.35			89.29	
Bank	3.46	2.67	2.74		2.88	2.34		17.14	5.97			4.13		3.82		2.48	2.73		2.32		2.25			82.86	
NBF	3.16	3	2.86		2.84	2.37		5.08	14.01			3.61		3.73		2.94	3.12		2.89		2.62			85.99	
Rest	3.08	3.89	3.18		2.54	2.9		2.85	2.86			11.3		3.95		2.93	2.88		2.88		2.88			88.7	
Trans	3.02	4.35	3.47		2.64	3.26		1.99	2.25			3.02		8.23		3.23	3.3		3.17		3.17			91.77	
Elec	2.91	4.12	4.05		2.5	3.32		1.33	1.85			2.34		3.34		8.58	4.7		4.79		3.75			91.42	
Comm	2.81	4.08	3.63		2.5	3.22		1.61	2.09			2.41		3.57		4.88	9.06		4.82		3.85			90.94	
Comp	2.85	4.5	3.9		2.25	3.27		1.33	1.91			2.4		3.39		4.99	4.86		8.79		4.51			91.21	
Media	2.81	4.51	3.76		2.23	3.46		1.36	1.83			2.59		3.62		4.2	4.2		4.82		10.02			89.98	
TO	80.05	108.97	97.56		71.14	86.6		49.16	59.73			75.99		101.7		96.49	91.89		91.38		84.27			2527.69	
NET	−9.2	16.54	6.18		−16.71	−2.68		−33.69	−26.26			−12.71		9.93		5.07	0.96		0.17		−5.71			93.62/90.27	

**Note:** The following table is the right part of the table above. Volatility connectedness within 28 sectors in the Chinese stock markets during the sample period from January 1, 2005 to May 31, 2023. The results are obtained by setting the predictive horizon *H* and the VAR lag order as 10 and 4 days, respectively. The sub-matrix from sector PP to sector Media reports the pairwise directional volatility connectedness between sector *i* and *j* (Eq. (12)), standing for the 10-day-ahead forecast error variance spilling from sector *j* to sector *i*. “From”, “To”, and “Net” correspond to the from-connectedness (Eq. (9)), to-connectedness (Eq. (10)), and net-connectedness (Eq. (11)) of sector *i*, respectively. The total connectedness and the numbers in the diagonal are h.

The Banking sector exhibits the highest self-volatility connectedness at 17.14%, with the Non-Bank Finance sector (NBF) following at 14.01%. These figures underscore the significant impact of internal shocks within the banking and financial sectors, emphasizing the critical need for stability to prevent industry disruption.

Most connectedness values are below 5%, indicating a general trend of systemic rather than individual sector dominance. However, the connectedness between the Mechanical (Mech) and Power and Utility (PU) sectors reaches 8.24%, a notable figure. This is likely due to the Mechanical industry’s substantial reliance on the stable power supply for production, highlighting the importance of the Power and Utility sectors’ development and energy supply stability for the Mechanical industry’s operations and growth. The total connectedness index stands at 90.27%, indicating a high degree of inter-industry risk spillover correlation.

### 4.2. To-degrees and from-degrees

**Fig 2:**
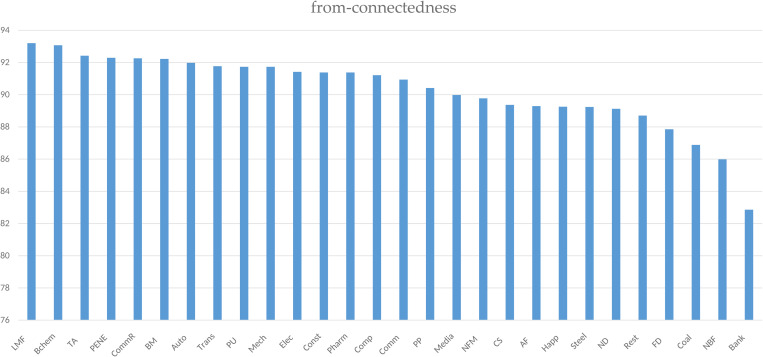
From-connectedness of sectors.

Fig 2 demonstrates constrained from-connectedness values ranging from 82.86% to 93.2%, with limited cross-sector variability. This narrow dispersion indicates homogeneous external shock sensitivity across industries. Specifically, Light Manufacturing (93.2%), Basic Chemicals (93.07%), and Textiles & Apparel (92.42%) exhibit the highest vulnerability, whereas Nonbank Financial (85.99%) and Banking (82.86%) sectors show relative resilience.

These findings suggest two policy implications: (1) developing unified risk mitigation strategies encompassing fiscal safeguards, sector-wide risk management training, and resilience-enhancing technological adoption; (2) implementing enhanced prudential oversight for financial institutions’ internal risk controls, given their lower systemic vulnerability.

**Fig 3:**
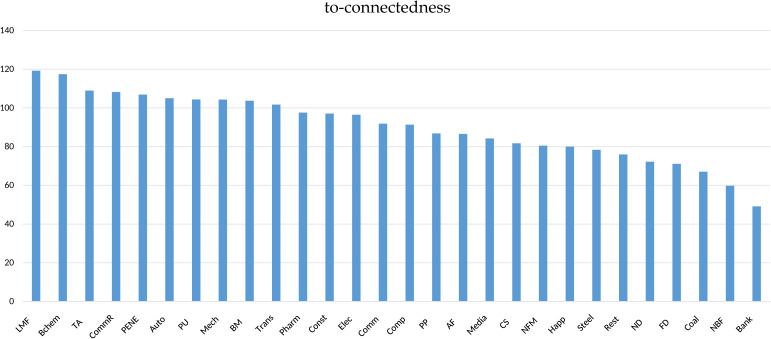
To-connectedness of sectors.

Fig 3 reveals substantial dispersion in to-connectedness values (49.16%−119.23%). indicating pronounced cross-sector heterogeneity in systemic risk emission. Light Manufacturing (119.23%), Basic Chemicals (117.42%), and Textiles & Apparel (108.97%) emerge as dominant risk propagators, while Nonbank Financial (59.73%) and Banking (49.16%) sectors demonstrate minimal outward transmission.

Two policy implications arise: (1) implementing enhanced monitoring frameworks for high-emission sectors (top decile >100%) through operational stability requirements and cascading risk containment measures; (2) fostering inter-sectoral risk-sharing mechanisms between financial institutions and real economy sectors to improve systemic risk absorption capacity.

**Fig 4:**
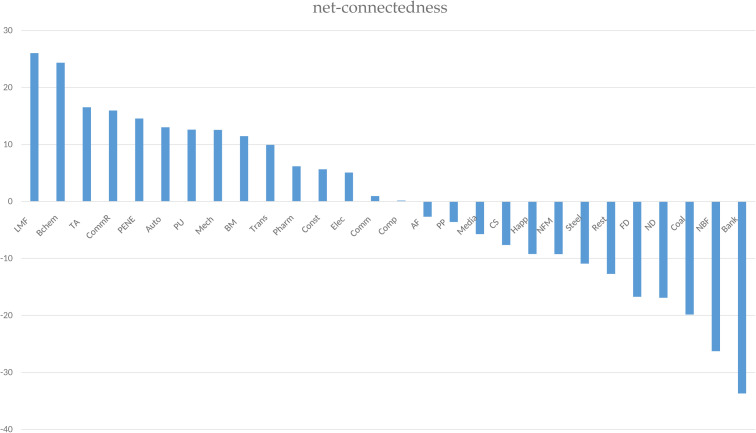
Net-connectedness.

Fig 4 indicates that the top three industries in terms of net-connectedness are Light Manufacturing, Basic Chemical, and Textile and Apparel, with the Non-Bank Finance and Banking sectors ranking at the bottom. The Non-Bank Finance and Banking sectors, being the primary recipients of risk, serve as the stabilizing force for national economic stability, playing a crucial role in the steady growth of the national economy. The study also reveals that the stability of the financial industry is contingent upon its own stability; financial instability would impair its ability to absorb and buffer risk impacts from the non-financial sectors, thereby jeopardizing economic stability.

Overall, 15 industries exhibit net-connectedness greater than 0, while 13 industries have net-connectedness below 0. Two industries, Light Manufacturing, and Basic Chemical, have network connectedness greater than 20. Seven industries fall within the range of 10–20 for network connectedness, including Textile and Apparel, Commercial Retail, Power Equipment and New Energy, Automobile, Power and Utilities, Mechanical Industry, and Mechanical Building Materials. Despite Commercial Retail not being a manufacturing industry, its interdependence with manufacturing—serving as the bridge between production and consumer demand—arrants its classification within the manufacturing-related sector.

Conversely, seven industries, including Bank, Non-Bank Financial, Coal Mining, National Defense, Food and Beverage, Real Estate, and Steel, have network connectedness of less than −10. Of these, Bank and Non-Bank Financial exhibit connectedness below −20, reflecting their strong financial attributes and classification as financial industries. In summary, the manufacturing industry is primarily an exporter of risk spillover, while the financial industry is the primary recipient.

Policymakers need to focus on the stability of the financial industry, as its stability directly affects its ability to absorb and buffer risks. Policies can include strengthening financial regulation, increasing capital adequacy and liquidity requirements for financial institutions, and establishing effective risk early warning mechanisms. For the manufacturing sector, policies can encourage risk reduction through technological innovation and industrial upgrading.

**Fig 5:**
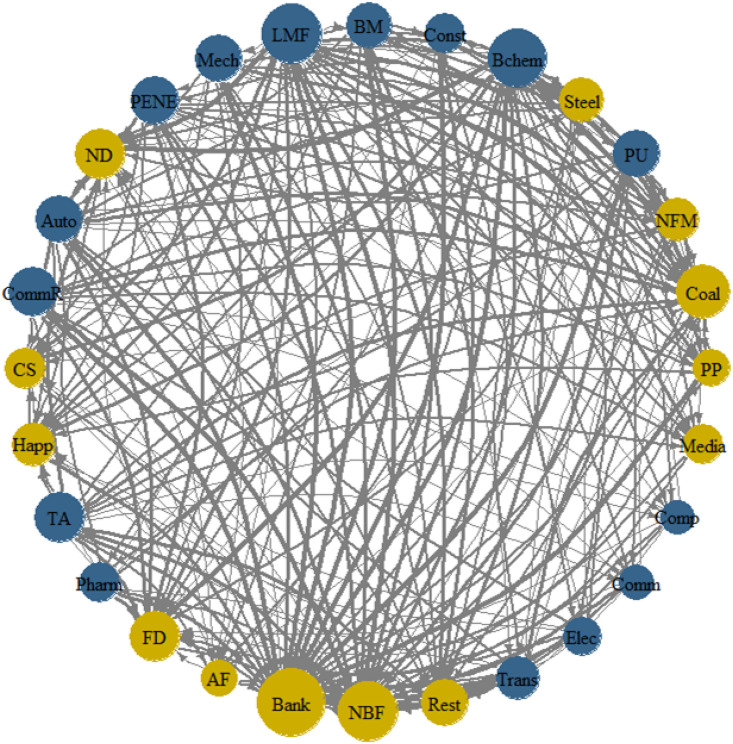
Network of NPDC. **Note:** Blue represents risk spillover, yellow signifies risk acceptance, and the size of the circles indicates the magnitude of spillover or acceptance.

NPDC (Net Pairwise Directional Connectedness) is an indicator used to assess the bidirectional connectedness between markets within a financial market system. Fig 5 illustrates that blue represents risk spillover, yellow signifies risk acceptance, and the size of the circles indicates the magnitude of spillover or acceptance. Light Manufacturing and Basic Chemical are the leading industries in terms of risk spillover, while Non-Bank Finance and Banking are the top industries in risk acceptance. For industries with high-risk spillover, policy should focus on their business models, risk management strategies, and the degree of interconnectedness with other sectors. For industries that accept risk, policy should encourage them to strengthen cooperation with other sectors to jointly address external risk challenges. Additionally, policymakers can use this graphic to identify potential risk transmission chains and take corresponding preventive measures.

**Fig 6:**
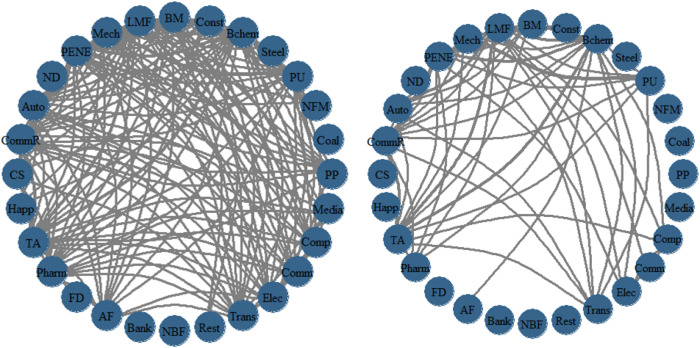
Network of PCI. (left: threshold sets 0.6; right: threshold sets 0.7). **Note:** PCI(Pairwise Connectedness Index) ,The thickness of the lines in the PCI graph measures the degree of connectivity between markets.

PCI (Pairwise Connectedness Index) is a metric used to assess the degree of interconnectedness between markets in a financial market system. The threshold parameter, denoted as *r*, sets the threshold for pairwise connectedness indexes. Fig 6 demonstrates this with two graphs: on the left, *r* = 0.6, and on the right, *r *= 0.7. The graphs reveal that many correlation coefficients fall within the range of 0.6 to 0.7. Both Non-Bank Finance and Banking show no connections with other industries, suggesting that the mutual spillover between the financial industry and other sectors is low. However, there is a high level of spillover among manufacturing-related industries. For the situation where risk spillover between the financial industry and other sectors is low, policymakers can explore ways to strengthen the connections between the financial industry and other sectors to promote better risk diversification and management. At the same time, in view of the higher risk spillover within the manufacturing sector, policy should encourage manufacturing enterprises to enhance internal management, technological innovation, and industrial upgrading to reduce the likelihood of risk spillover. Additionally, policymakers can use threshold parameters to set trigger conditions for risk management to ensure that timely measures can be taken for intervention before risks occur.

## 5. Benchmark results II: Dynamic (Rolling-Sample) connectedness

Static industry connectedness is calculated based on data from the entire sample period, reflecting the average connectedness between industries over a fixed time span. Dynamic industry connectedness, on the other hand, is computed using a rolling window technique, which involves incrementally moving the window across the sample period and calculating the connectedness for each window. Static connectedness aids in formulating long-term industrial policies, while dynamic connectedness provides a basis for addressing short-term market fluctuations. For instance, during a financial crisis, dynamic connectedness can reveal which industries are the primary transmitters and recipients of risk, thus offering guidance for policy interventions.

### 5.1. Evolution of total connectedness

The evolution of the Total Connectedness Index (TCI) within sectors from January 1, 2005, to May 31, 2023, is presented. The TCI is derived from a rolling window analysis with a window size of 200 days, a moving step of one day, a lag order of 4, and a predictive horizon of 10 days.

**Fig 7:**
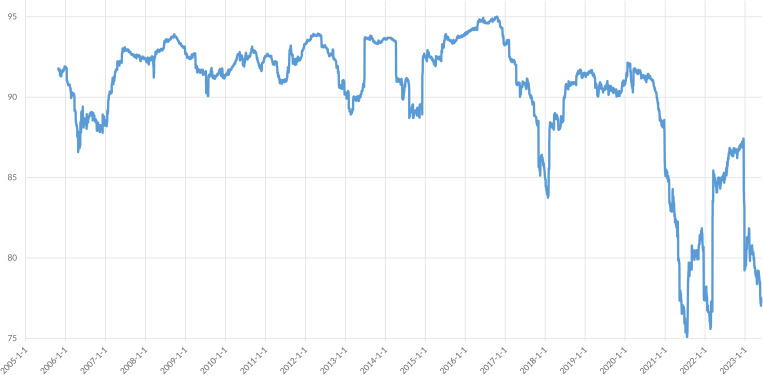
Total Connectedness Index.

The Total Connectedness Index (TCI) serves as a comprehensive metric for evaluating the overall correlation within the financial market system. It considers the correlation between all industries and is an indicator of the system’s overall relevance. A higher TCI value indicates a stronger connection within the system and a greater degree of information transmission and influence between industries. Fig 7 depicts the dynamic behavior of the TCI, which exhibits volatility with fluctuations ranging between 75.09% and 95.00%. These fluctuations reflect significant changes in the industry correlation within China’s stock market and major economic shocks.

The TCI exhibits a pronounced “near-crisis” characteristic, characterized by a gradual build-up of risk before a crisis and a sharp increase in financial market volatility during the crisis, leading to a significant increase in risk spillover until it reaches its peak. Following a crisis, the risk gradually subsides and the TCI stabilizes with slight fluctuations. This behavior aligns with the findings of Diebold and Yilmaz (2012) [13], indicating that the TCI can act as an “early warning system” for impending crises and track the progression of existing crises.

The performance of the market and risk spillover indexes before and after the 2008 U.S. financial crisis demonstrates this “near-crisis” feature. From 2007 to 2009, the overall risk spillover index increased, peaking during the crisis and declining afterward. This trend also captured the stock market crash from November 2007 to October 2008, where the Shanghai Composite Index fell from 6124 to 1664 points, marking a 72.8% decline over 12 months. The “near-crisis” characteristic of the TCI provides valuable insights for policymakers. The gradual build-up of risk before a crisis suggests that early detection of rising TCI values can serve as a crucial signal for potential systemic instability. Policymakers should focus on developing robust monitoring tools that can identify these early warning signs and implement timely interventions to prevent the escalation of risks. For example, during periods of rising TCI, regulatory authorities could enforce stricter capital requirements or liquidity standards for financial institutions to ensure they are better prepared to handle increased volatility and spillovers. This proactive approach can help in mitigating the severity of crises and reducing the likelihood of systemic failures.

The “near-crisis” feature has recurred multiple times. The European debt crisis (2011–2015) saw a rise in the industry-wide risk spillover index, as did the interbank liquidity crisis in June 2013, the stock market crash from June 2015 to January 2016, and the market turmoil following Vladimir Putin’s announcement of a military operation in the Donbas region in February 2022. Each of these events was accompanied by a significant increase in the TCI, albeit with varying durations.

The initial outbreak of COVID-19 in Wuhan (December 2019) and subsequent lockdown measures (January 2020) triggered a transient surge in the Total Connectedness Index (TCI), followed by a gradual decline. This pattern indicates limited persistence of pandemic-induced effects on systemic risk transmission. Two key drivers of the temporary connectivity escalation are: (1) investor panic-induced cross-sector volatility spillovers, and (2) supply chain disruptions across critical manufacturing nodes.

The economic factors contributing to the decline in connectedness during the epidemic may include the gradual recovery of economic activities, industry differentiation, changes in investor sentiment, and policy regulation. As the economy stabilizes, the connectedness between industries may decrease, reflecting the differing impacts of the epidemic on various sectors.

### 5.2. Evolution of sector connectedness

We analyze both total system-wide and total directional (to and from) connectedness. From a dynamic perspective, our analysis confirms the law of static presentation. The numerical change of from-connectedness is relatively small, and most values decrease after 2021. In contrast, the to-connectedness values exhibit significant variation, with the total directional (to and from) connectedness being highest for Light Manufacturing, Base Chemical, and Textile and Apparel, and lowest for Non-Bank Finance and Banking.

**Fig 8:**
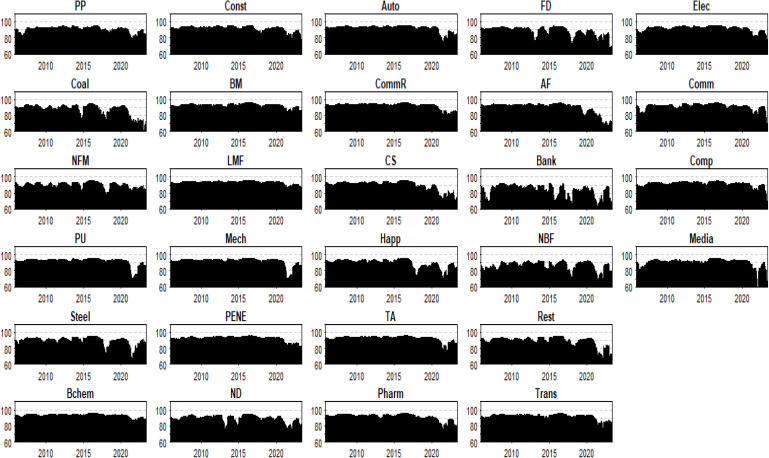
Evolution of from-connectedness.

Fig 8 demonstrates converging from-connectedness patterns, revealing that systemic shocks propagate through cross-sectoral risk spillover effects (analogous to butterfly-effect mechanisms). This homogenization of risk exposure indicates uniform sectoral vulnerability to external disturbances, potentially exacerbating systemic risk accumulation.

Three policy imperatives emerge: (1) Implementing system-wide risk monitoring frameworks instead of sector-specific approaches; (2) Developing cross-sectoral stress testing protocols to evaluate financial system resilience; (3) Establishing regulatory architectures mandating industry diversification requirements and resilience benchmarking.

**Fig 9:**
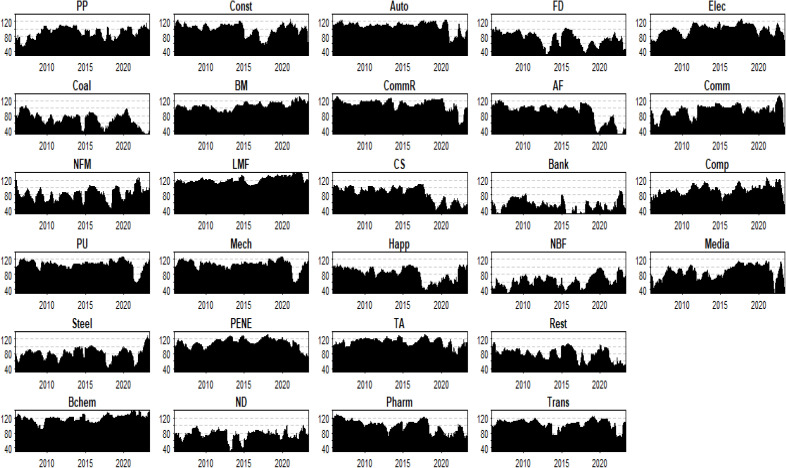
Evolution of to-connectedness.

Fig 9 indicates that Light Manufacturing, Base Chemical, Textile and Apparel, Commercial Retail, Power Equipment and New Energy, Automobile, Power and Utilities, Mechanical Industry, and Building Materials exhibit a higher degree of risk spillover effects. These sectors predominantly fall under the umbrella term of “Manufacturing Attributes,” which includes infrastructure and operations, as well as the TMT sector (Technology, Media, and Telecommunications). In contrast, sectors such as Agriculture and Forestry, Media, Customer Service, Non-Ferrous Metals, Household Appliances, Steel, Real Estate, National Defense, Food and Drink, Coal Mining, Non-Bank Finance, and Banking demonstrate weaker overall risk spillover effects, generally classified under the “Service Attributes” category.

In summary, Fig 9 clearly depicts the disparities in risk transmission intensity among different industries, highlighting the distinct dynamics between industries with “Manufacturing Attributes” and those with “Service Attributes” when exposed to risk spillovers. The higher degree of risk spillover effects observed in manufacturing-related sectors can be attributed to their extensive supply chains and interconnected operations, which make them more susceptible to disruptions and the propagation of shocks. Policymakers should consider implementing targeted measures to enhance the resilience of these sectors, such as promoting the adoption of advanced manufacturing technologies or supporting the development of more robust supply chain networks. For service-oriented sectors with weaker risk spillover effects, the focus could be on ensuring that they maintain adequate liquidity and operational flexibility to withstand potential shocks.

**Fig 10:**
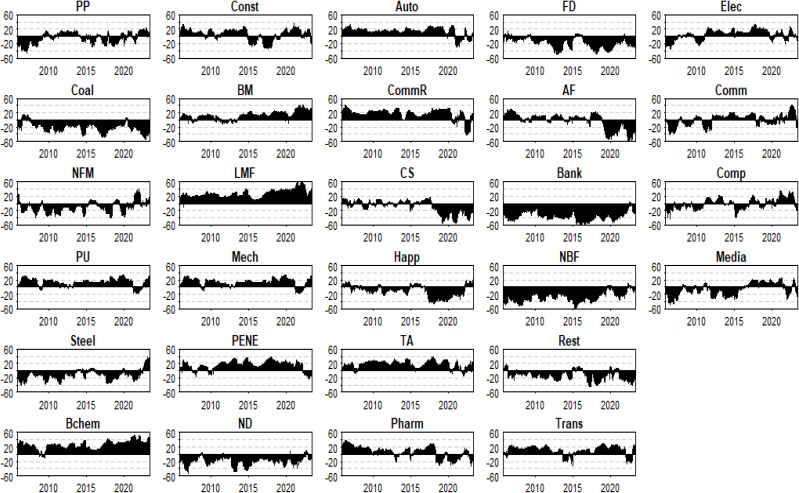
Evolution of net-connectedness.

Fig 10 reveals that industries such as Light Manufacturing, Base Chemical, Textile and Apparel, Commercial Retail, Power Equipment and New Energy, Automobile, Power and Utilities, Mechanical Industry, Building Materials, and Transportation are predominantly net exporters of risk spillovers. Industries such as Electronics, Pharmaceuticals, Construction, Communications, Computer, Agriculture and Forestry, Petroleum and Petrochemicals, Media, and Customer Service exhibit neither a net sender nor net receiver status over time. This indicates that due to external emergencies and industrial restructuring, there is a structural mutation in the spillover attributes of the industry. Ignoring this transformation in the risk contagion process may lead to the failure of risk prevention and control policies. Moreover, industries such as Non-Ferrous Metals, Household Appliances, Steel, Real Estate, National Defense, Food and Drink, Coal Mining, Non-Bank Finance, and Banking are predominantly net receivers of connectedness. Non-Bank Finance and Banking are pure receivers and are greatly affected, which aligns with the static conclusions.

Fig 10 reveals that specific risks can have heterogeneous impacts on different industries, as evidenced by changes in net-connectedness before and after these events. For example, during the COVID-19 pandemic, the pharmaceutical industry shifted from being a net exporter of risk to a net importer. During the Russia-Ukraine conflict, China’s national defense industry shifted from being a net importer to a net exporter and then back to a net importer.

The dynamic nature of risk spillovers during the COVID-19 pandemic and the Russia-Ukraine conflict underscores the necessity for continuous monitoring and adaptive policy frameworks. Given the heterogeneous impacts of these events on different industries, such as the pharmaceutical industry during the pandemic and China’s national defense industry during the conflict, it is crucial to establish a robust inter-industry risk monitoring framework. This framework should track, in real-time, the risk spillover and reception across various sectors, with a particular focus on industries that exhibit significant changes in their net-connectedness during and after risk events. For instance, during the Russia-Ukraine conflict, China’s national defense industry initially became a net exporter of risk due to increased global demand for military equipment, driven by heightened geopolitical tensions. However, as the conflict continued, international demand for military trade declined, while China’s own defense construction needs grew, and the supply of some key components and technologies remained uncertain, causing the industry to revert to a net importer. Similarly, the pharmaceutical industry saw a surge in demand for vaccines and medical supplies during the pandemic, making it a net exporter of risk. As the pandemic waned, demand for COVID-19-related products declined, competition intensified, and the industry transitioned back to a net importer.

In conclusion, the experimental results presented in Figs 2-10 provide a comprehensive view of the risk spillover landscape in China’s economy. These findings contribute to a better understanding of systemic risk and its potential implications for financial stability. The insights gained from this analysis can guide policymakers in formulating effective risk management strategies and can inform the design of robust financial regulatory frameworks.

### 5.3. Robustness tests

We employ the elastic network method to estimate the coefficients of the Vector Autoregression (VAR) model and subsequently utilize the method proposed by Diebold and Yilmaz (2012) [13] to compute the connectedness. The static connectedness results are essentially uniform. Generally, the diagonal elements in the connectedness matrix are larger than those obtained using the LASSO method, with the risk spillover and acceptance industries remaining largely unaltered. The risk connectedness values exhibit minimal changes, and the rankings shift slightly but remain within a narrow range, maintaining the original characteristics.

By employing a lag order of 2, a prediction horizon of 10, and a rolling window width of 200, and using the LASSO estimation method, the static and dynamic connectedness calculations exhibit only minor discrepancies from the results obtained with a lag order of 4, a prediction horizon of 6, and a rolling window width of 200, using the same LASSO estimation method. This consistency aligns with the original experimental outcomes.

From the experimental results presented, it is evident that the outcomes derived from various estimation methods and parameter configurations are predominantly consistent, indicating the stability of the research findings.

## 6. Conclusion

### 6.1. Research results

Building upon the vector autoregressive (VAR) model and variance decomposition, a volatility spillover network has been established to quantify the correlation of volatility among sectors in China’s stock market. Initially, we constructed a correlation matrix based on the full sample data, providing a comprehensive view of the total correlation degree between industries, encompassing the correlation degree from, to, and the net correlation degree. The empirical findings reveal that the largest element within each row of the correlation matrix resides on the diagonal, indicating that the primary risk contributor to each sector is its own volatility. Fifteen industries, including Light Manufacturing, Base Chemical, and Textile and Apparel, are identified as risk passers, while 13 sectors, such as Non-Bank Finance and Banking, are categorized as risk takers. In essence, the manufacturing-attribute industries tend to be risk exporters, while the resource and service-attribute industries are more likely to be risk receivers.

Subsequently, the rolling window method is employed for dynamic analysis. The study reveals that the Total Connectedness Index (TCI) fluctuates in tandem with stock market volatility and spikes during major events that precipitate a sharp decline in the market, such as the global financial crisis, the European debt crisis, instances of ‘money shortage,’ stock market crashes, the Sino-U.S. trade war, and the Russia-Ukraine conflict. The outbreak of the novel coronavirus in 2020, however, exceeded expectations, with the connectedness initially rising slightly before declining. Similarly, the Russia-Ukraine conflict exhibited a comparable impact on risk spillover.

Finally, the robustness of the model is tested by utilizing various VAR coefficient estimation methods and different parameters (lag order p, prediction range h, and rolling window width W). The findings indicate that the Generalized Variance Decomposition (GVD) method, based on diverse coefficient estimation methods, the VAR model, and the rolling window method, is insensitive to changes in model parameters. This suggests that our results are robust and reliable. Our research not only elucidates the spillover effects between industries in China but also provides a profound understanding of the risk contagion model within China’s stock market.

### 6.2. Managerial implications

For industries that export systemic risk (such as light manufacturing and basic chemicals), the findings suggest the necessity for a three-pronged strategic adjustment. First, enhancing supply chain resilience by constructing multi-tiered supplier networks and selecting 3–5 certified partners for critical inputs, especially through mineral co-development agreements under the Belt and Road Initiative to mitigate geopolitical risks. Second, establishing intelligent risk monitoring systems that integrate IoT-enabled operational data (such as equipment utilization and inventory turnover) with market indicators (like commodity futures and the Purchasing Managers’ Index), employing machine learning algorithms to forecast volatility spillovers. In parallel, collaborating with fintech partners to develop stress-testing platforms that simulate cross-industry contagion pathways. Third, manufacturing clusters must institutionalize risk-sharing mechanisms; for example, the Yangtze River Delta alliance has established stabilization funds and developed volatility swap derivatives to hedge sector-specific shocks. Meanwhile, embedding Environmental, Social, and Governance (ESG) metrics into supplier evaluation systems and financing low-carbon retrofits through the issuance of green asset-backed securities (ABS) to cushion the impact of environmental regulations.

For industries that receive systemic risk (such as non-bank finance and banking), the evidence indicates the need for adaptive risk governance frameworks. Financial institutions should implement dynamic capital buffers that automatically increase liquidity coverage ratios by 10% when the Total Connectedness Index (TCI) exceeds one standard deviation. It is essential to utilize AI-driven surveillance systems that integrate cross-agency data (such as tax records and customs logs) and combine Natural Language Processing (NLP) analysis of corporate disclosures to generate alerts for critical thresholds (e.g., supplier concentration exceeding 30%). Stress-testing protocols need to be redesigned to incorporate network contagion effects, simulating multi-channel shocks, including 10-day supply chain disruptions and ±20% commodity price fluctuations. To alleviate cross-industry spillovers, developing volatility index futures and cash flow swaps that link manufacturing revenues with financial asset returns can provide natural hedging instruments.

### 6.3. Policy implications

The empirical findings validate a comprehensive four-tier regulatory framework designed to enhance systemic stability through coordinated risk management. The first tier consists of automatic stabilization protocols that provide foundational safeguards. The second tier implements progressive TCI-based constraints, including sectoral credit ceilings of 15% when manufacturing TCI exceeds historical 90th percentile thresholds, alongside a three-phase alert system: exposure audits trigger at TCI ≥ 60th percentile, cross-industry guarantee caps activate at TCI ≥ 75th percentile, and transaction levies apply at TCI ≥ 90th percentile.

The third tier introduces differentiated supervision mechanisms tailored to specific sectors. Export-oriented industries receive 3%–5% VAT rebates for digitalization compliance but must meet supply chain resilience reporting requirements. Systemically important financial institutions face stricter measures, including correlation-adjusted risk weights that increase by 5% for every 10% rise in interlinkages, alongside mandatory digital twin stress testing.

The fourth tier focuses on institutional infrastructure upgrades, notably a national risk monitoring platform integrating over 15,000 corporate nodes for real-time contagion mapping. Additionally, legislative reforms empower the PBOC to intervene when cross-sector correlations reach ρ ≥ 0.7.

Finally, global coordination instruments strengthen cross-border safeguards, including a US$20 billion BRI stabilization reserve and multinational commodity buffers triggered by CRB Index fluctuations beyond ±15% volatility bands. Together, this multi-layered architecture systematically enhances stability through risk containment, resilience-building, and international safeguards.

### 6.4. Limitations of the research

While our research provides valuable insights into the volatility spillover dynamics in China’s stock market, two limitations should be acknowledged:

The study is based on data from China’s stock market, which may not fully capture the dynamics of global financial markets. Future research could extend the analysis to other major economies to validate the findings and enhance the generalizability of the results.

The VAR model and variance decomposition methods rely on assumptions such as linearity and normality, which may not always hold in real-world scenarios, especially during extreme events. Exploring non-linear models and alternative statistical methods could provide a more nuanced understanding of volatility spillovers.

## Supporting information

S1 File Dataset used for analysis. (ZIP)
